# A Misalignment Optical Guiding Module for Power Generation Enhancement of Fixed-Type Photovoltaic Systems

**DOI:** 10.3390/mi10100687

**Published:** 2019-10-11

**Authors:** Cheng-Tang Pan, Chung-Kun Yen, Shao-Yu Wang, Pei-Yuan Sun, Sin-Yu Huang, Yeong-Maw Hwang, Zong-Hsin Liu, Li-Ming Chu, Zheng-Yu Hoe

**Affiliations:** 1 Department of Mechanical and Electro-Mechanical Engineering, National Sun Yat-sen University, Kaohsiung 80424, Taiwan; pan@mem.nsysu.edu.tw (C.-T.P.); alden0113@gmail.com (C.-K.Y.); sywang@mem.nsysu.edu.tw (S.-Y.W.); iverson2559@gmail.com (P.-Y.S.); whu924210@gmail.com (S.-Y.H.); ymhwang@mail.nsysu.edu.tw (Y.-M.H.); 2 Institute of Medical Science and Technology, National Sun Yat-sen University, Kaohsiung 80424, Taiwan; 3 Metal Industries Research and Development Centre, Kaohsiung 81160, Taiwan; inzon513@mail.mirdc.org.tw; 4 Interdisciplinary Program of Green and Information Technology, National Taitung University, Taitung 95092, Taiwan; lmchu@nttu.edu.tw; 5 Department of Physical Medicine and Rehabilitation, Kaohsiung Veterans General Hospital, Kaohsiung 81362, Taiwan

**Keywords:** misalignment light-guiding module, non-axisymmetric compound parabolic curve, freeform surface collimator

## Abstract

This study presents a misalignment light-guiding module to increase the effectiveness of absorbing light. For a general fixed-type photovoltaic (PV) panel, the misalignment light decreases the efficiency of the system. A solar tracking system was installed for obtaining higher power generation. However, the cost of the PV system and maintenance was 5–10 times higher than the general type. In this study, this module is composed of an array of misalignment light-guiding units that consist of a non-axisymmetric compound parabolic curve (NACPC) and a freeform surface collimator. The NACPC efficiently collects the misalignment light within ±30° and guides the light to the collimator. The light has a better uniformity and smaller angle at the exit aperture. The simulation results show that the optical efficiency of the unit was above 70% when the misalignment angle was smaller than 20°. The experimental results show that the power generation of the light-guiding unit was 1.8 times higher than the naked PV panel.

## 1. Introduction

In the last few decades, there were significant changes in the use of the world’s energy resources. Governments, industries, and academic institutions sought to find alternative sources of energy and improve energy efficiency. Among all alternative sources of energy, solar photovoltaic (PV) approaches attract significant attention. PV is a simple way to generate electricity from solar radiation.

The earth receives approximately 1 kW/m^2^ of solar irradiation in a day [1]. Abbot showed that this amount of irradiation could generate around 85,000 TW and estimated that the current global energy consumption was about 15 TW [2]. However, the cost of PV systems is high, and this is a barrier in competing with conventional electricity technologies. Therefore, studies on solar concentrators are becoming more and more common. These devices can concentrate solar radiation onto a small area, and the size of the PV cell can be reduced. Also, the cost of the PV system can be reduced. A previous study indicated a cost reduction of 40% using a solar concentrator with a geometrical concentration ratio of 2.45 [3]. Also, a normal PV cell may have higher conversion efficiency under concentrated solar radiation [4].

Solar concentrators [5] also generate heat on the PV cell. The increasing temperature causes the efficiency of the PV cell to reduce [6]. A low-concentration PV system was able to solve this problem at a low cost. The compound parabolic curve (CPC) is a well-known low-concentration solar concentrator. The CPC was proposed by Winston and further developed by Welford, mainly in the 1970s [7,8]. A wider angular region of the sky can be concentrated by the CPC, including a substantial portion of the diffuse radiation. This means that the CPC can be used with a less precise tracking system. Furthermore, its simple appearance received great interest in building-integrated photovoltaic and thermal applications [9]. There are two basic types of CPC: mirror or solid. Different types of CPC designs incorporate different features [10,11,12]. The CPC can either be used as a three-dimensional rotational symmetry concentrator or as a CPC trough concentrator [13]. Additionally, the solid CPC employs total internal reflection (TIR) and may have a potential high optical efficiency. A concentrator with a geometrical concentration ratio of two was developed in Sydney, Australia, and the efficiency was as high as 94% (the efficiency is defined as light collection efficiency, which is defined as the ratio of the input and output incident sun light) [14]. However, the solid CPC consumes lots of material. A truncated CPC (T-CPC) was studied, and it was found to have a minor reduction in optical efficiency by reducing the length of the CPC [15]. A lens-walled CPC was also developed [16]. A novel type of solar concentrator called a rotationally asymmetrical compound parabolic concentrator (RACPC) was also presented [17].

In this study, a misalignment light-guiding module was investigated. Owing to the characteristics of CPCs, the CPC was chosen to become a part of misalignment light-guiding module with the responsibility of collecting a wider angular region of the sky. For higher area utilization, the CPC was transformed into a non-axisymmetric compound parabolic curve (NACPC). A freeform surface collimator was applied to align the light from the NACPC and provide a better uniformity. The NACPC and the freeform surface collimator constituted the misalignment light-guiding unit, and the unit was put in an array attached to the PV panel to obtain a misalignment light-guiding module. 

## 2. Experimental Methods

### 2.1. Non-Axisymmetric Compound Parabolic Curve Design

**Figure 1:**
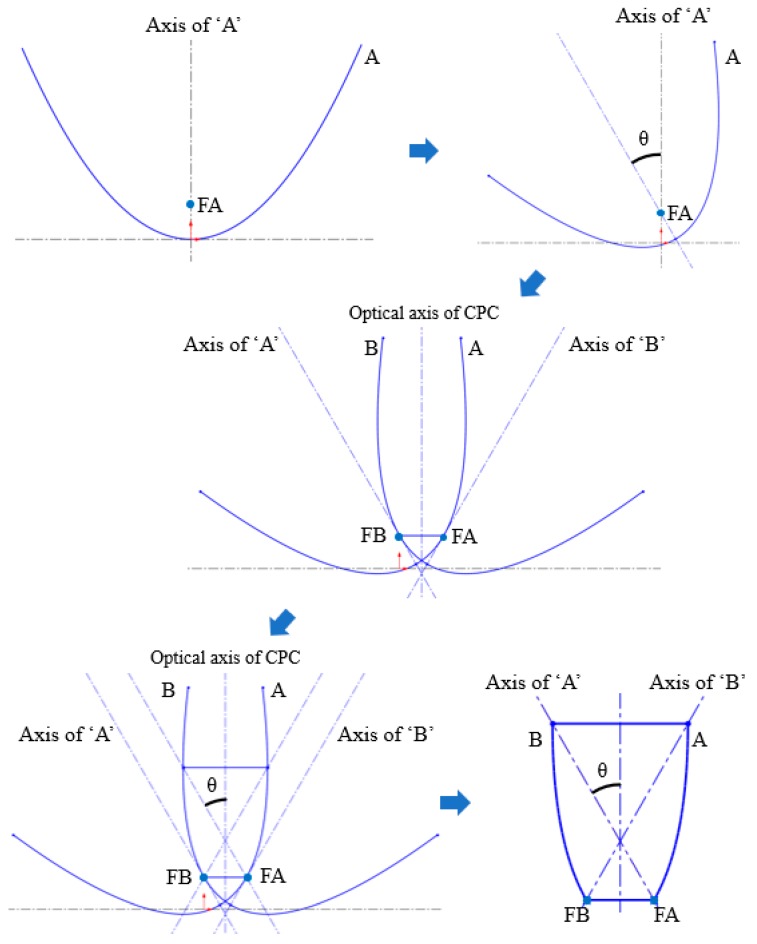
The steps for constructing a compound parabolic curve (CPC).

**Figure 2:**
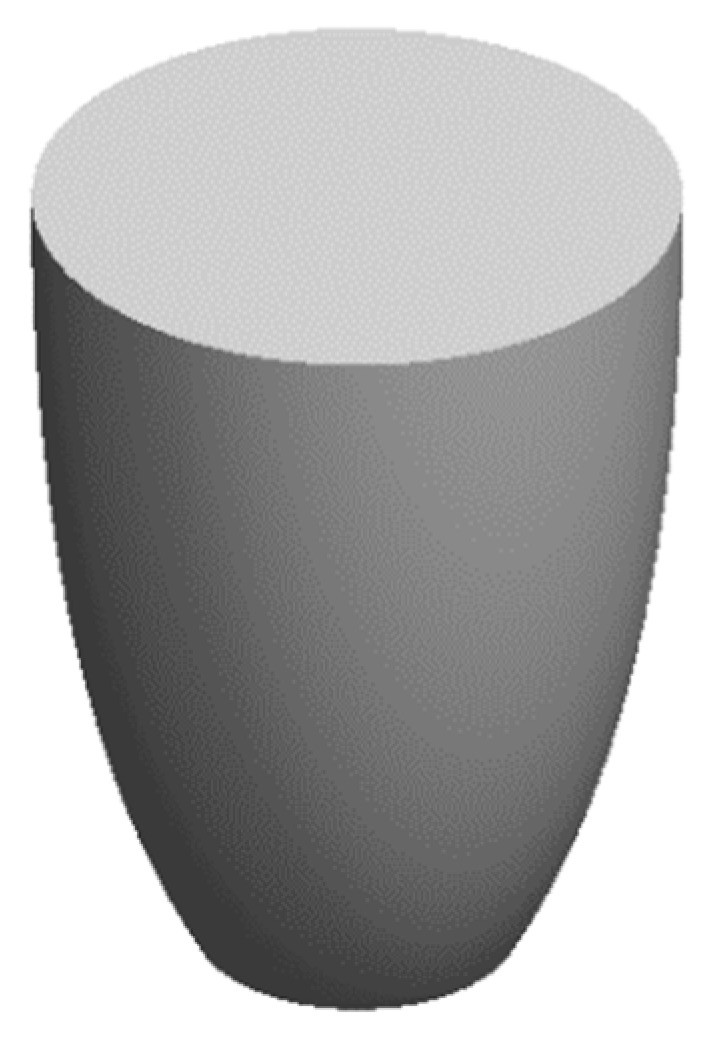
The appearance of a CPC.

**Figure 3:**
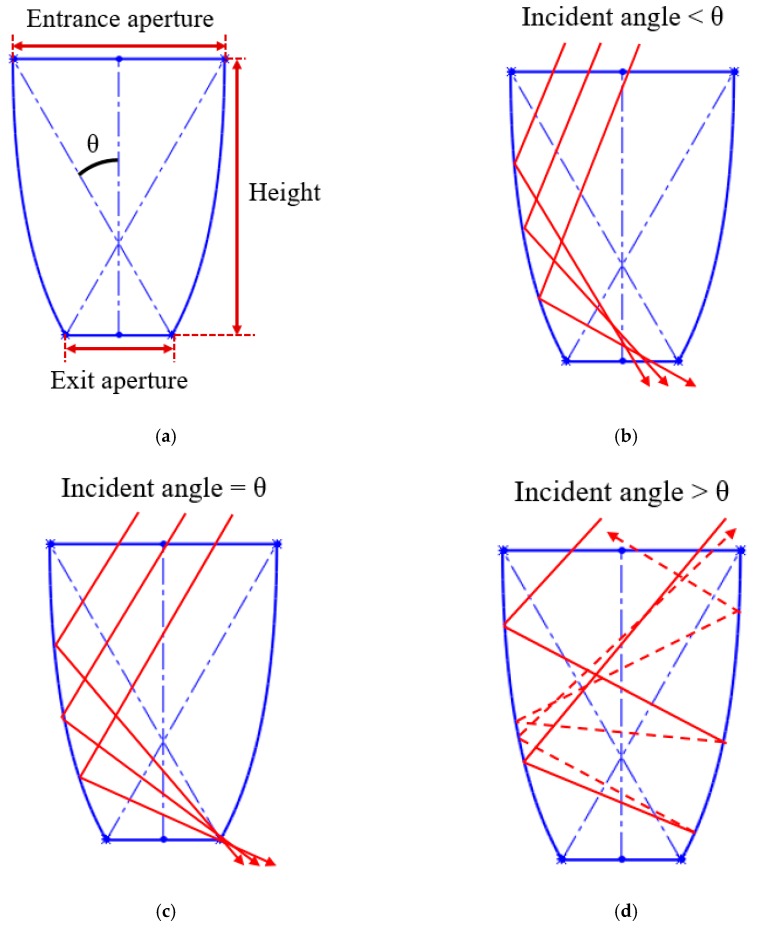
The principle behind the CPC, (**a**) definition of the half-acceptance angle, (**b**) incident angle smaller than the half-acceptance angle, (**c**) incident angle equal to the half-acceptance angle, (**d**) incident angle larger than the half-acceptance angle.

**Figure 4:**
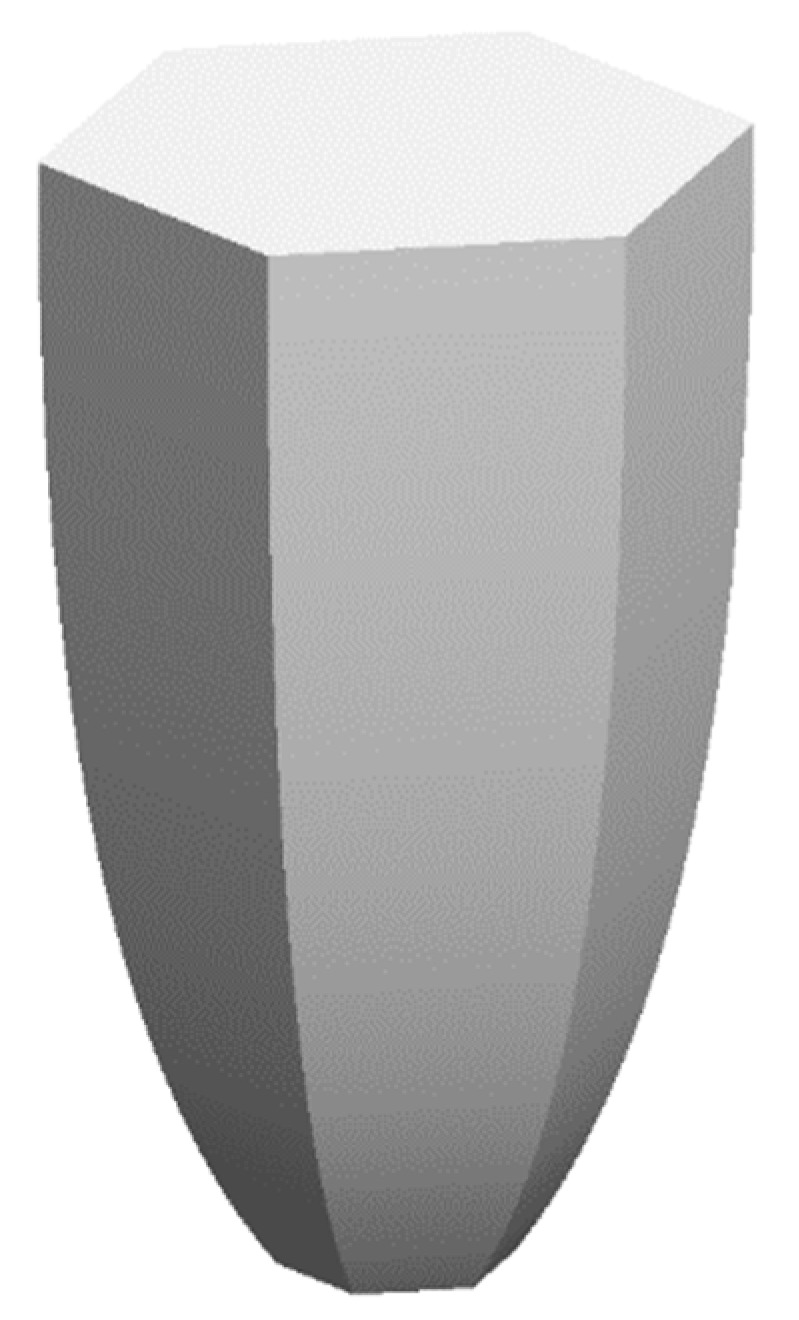
The appearance of a non-axisymmetric compound parabolic curve (NACPC).

The design basis of the NACPC came from the improvement of the CPC. The CPC was designed by two parabolas with different rotating angles, as shown in Figure 1. Firstly, two identical parabolas were applied for the CPC. One was called parabola A, and the other was called parabola B. Parabola A rotated in a counterclockwise manner along with its focal point, and parabola B rotated in a clockwise manner along with its focal point. Focal point FA was located on parabola B, and focal point FB was located on parabola A. Then, the unnecessary parts of the parabolas were truncated. These two parabolas were rotated to generate a three-dimensional structure based on an asymmetry axis, as shown in Figure 2. This structure was either designed as a mirror or solid type. For the mirror type, the CPC was in the form of a shell with a thin film coating on the surface, where the surface resembled a mirror with total internal reflection. For the solid type, the CPC was in the form of a bulk material with reflection and refraction. One kind of wavelength (546.1 nm) was used in the analyses to simplify the complexity of the simulation. For the mirror type, there was no effect on the light entering the aperture of CPC. However, for the solid type, there was refraction when the light entered the aperture of CPC. Therefore, the angle between the normal and the light entering the CPC was regarded as the incident angle of light in this study. The angle after refraction was the incident angle of light. Angle *θ* was defined as the half-acceptance angle, as shown in Figure 3a. When the incident angle of light was smaller than the half-acceptance angle, the light smoothly went out through the exit aperture, as shown in Figure 3b. When the incident angle of light was equal to the half-acceptance angle, the light went out at focal point FA or FB, as shown in Figure 3c. When the incident angle of light was larger than the half-acceptance angle, the light was reflected by the wall several times. Then, the light went out through the entrance aperture, as shown in Figure 3d. Circular CPCs resulted in several gaps between each CPC unit when the CPCs were arranged in the form of an array. Therefore, the CPCs could not be arranged in an array. These gaps caused a low fill factor and a significant decrease in efficiency. Thus, the NACPC design was applied to improve these issues, as shown in Figure 4. The hexagonal shape arrangement enhanced the fill factor, as well as the sunlight collection area and efficiency.

### 2.2. Freeform Surface Collimator Design

**Figure 5:**
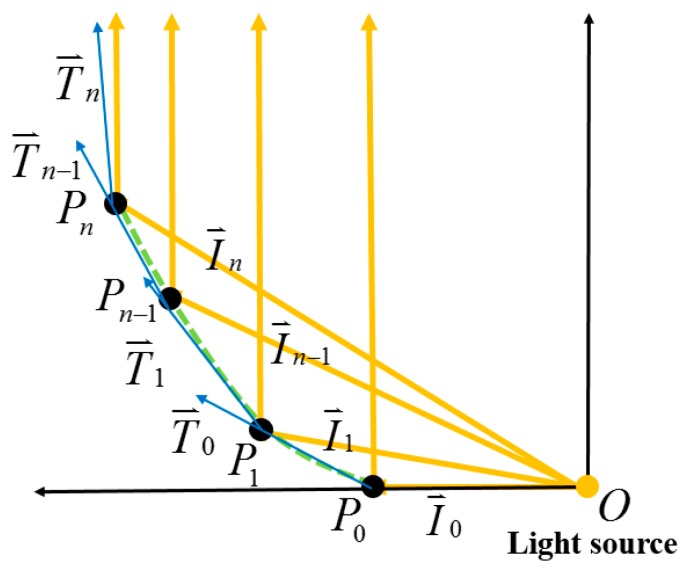
The process for constructing the freeform reflection surface.

**Figure 6:**
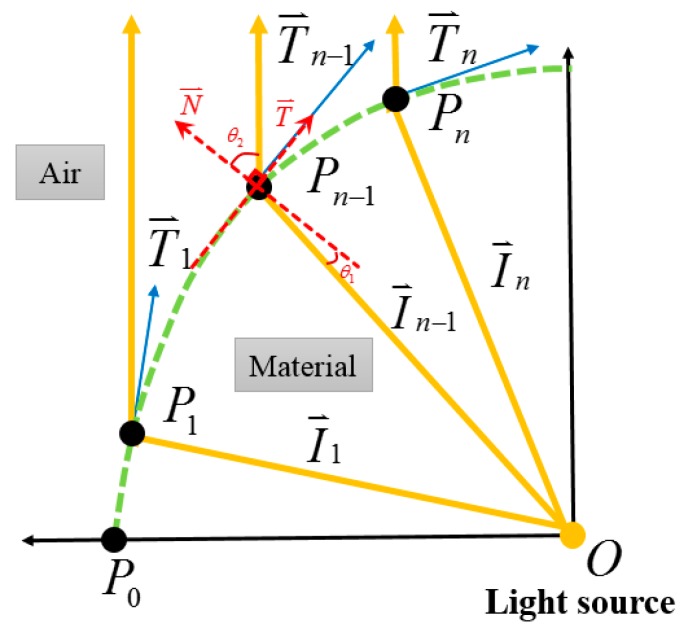
The process for constructing the freeform refraction surface.

In order to better align the light from the CPC, a collimator was included in the module, and the freeform surface approach was chosen to construct the collimator. The traditional approach is to couple pieces of lenses, resulting in the whole system having a higher cost and needing more space. However, the freeform surface approach allows forming each surface separately before constructing a lens. The space and cost were, thus, saved. Firstly, the light from the exit aperture of the NACPC was regarded as the point light source. Two kinds of design rules were chosen depending on the need. One involved constructing a reflection surface, and the other involved constructing a refraction surface. Figure 5 and Figure 6 show the processes for the free-form reflection surface and refraction surface, respectively. The original point $P_{0}$ and the vector of the collimated light were decided. Also, the direction vector $\vec{I_{0}}$ was obtained. The tangent vector $\vec{T_{0}}$ at the point $P_{0}$ could be calculated using Snell’s Law. Snell’s Law is described in Equation (1). The tangent vector $\vec{T_{0}}$ and the direction vector $\vec{I_{1}}$ intersected at a point $P_{1}$. The tangent vector $\vec{T_{1}}$ at point $P_{1}$ was also calculated using Snell’s Law. Then, the process was repeated to obtain all direction vectors $\vec{I_{n}}$, tangent vectors $\vec{T_{n}}$, and points $P_{n}$. Finally, each point $P_{0}$, $P_{1}$…$P_{n}$ could be drawn in a line to finish the freeform surface collimator [18].

(1) $${\left[n_{1}{}^{2}+n_{2}{}^{2}-2n_{1}n_{2}(\vec{O}\cdot \vec{I})\right]}^{\frac{1}{2}}\cdot \vec{N}=n_{2}\vec{O}-n_{1}\vec{I},$$

where $n_{1}$ and $n_{2}$ are the refractive index of the air and the collimator, respectively. $\vec{O}$, $\vec{I}$, and $\vec{N}$ are the exit vector, the incidence vector, and the normal vector, respectively.

**Figure 7:**
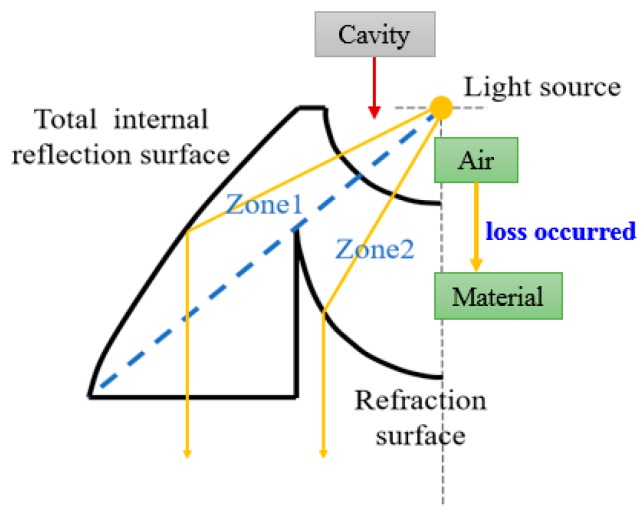
The concept of the total internal reflection (TIR) collimator.

Using this approach and other collimators [19], four kinds of collimator were constructed: a TIR type, an elliptical type, and two variants based on these two types. Figure 7 shows the concept of the TIR collimator, with two zones included in the design. In Zone 1, the light goes through the spherical surface first, propagates inside the material, and then reflects internally on the surface. Therefore, the light exits the TIR collimator in parallel with the axis. In Zone 2, the light also goes through the spherical surface first, propagates inside the material, and then refracts on the surface. Finally, the light exits the TIR collimator in parallel with the axis. This collimator design can make sure that the output light propagates in parallel with the axis.

**Figure 8:**
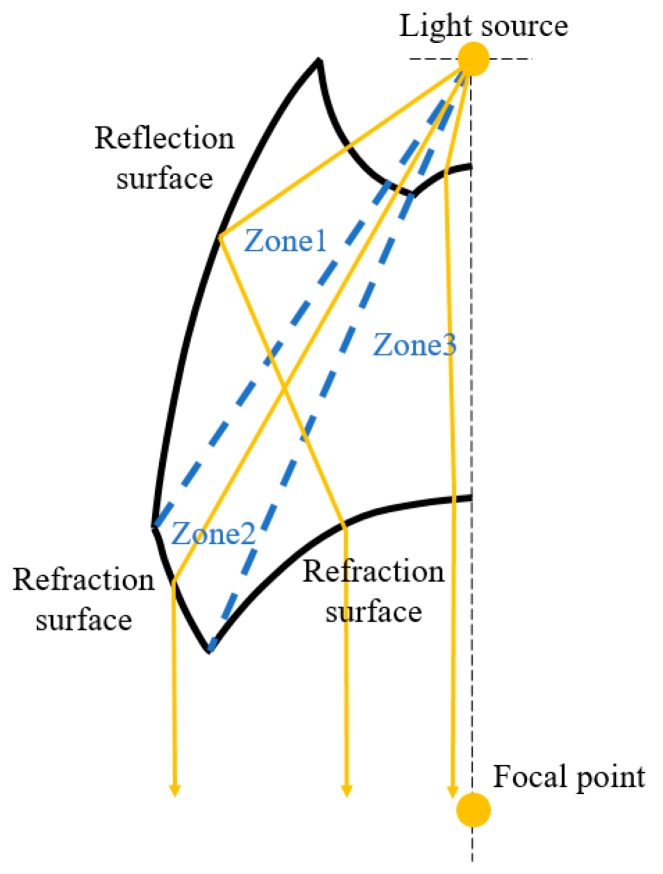
The concept of the elliptical collimator.

The second type was an elliptical collimator. The concept of the elliptical collimator is shown in Figure 8, with three zones included in the design. In Zone 1, the light goes through the spherical surface and reflects toward the focal point via the surface. Then, the light refracts at the refraction surface, resulting in the light exiting the elliptical collimator in parallel with the axis. In Zone 2, the light also goes through the spherical surface and refracts at the refraction surface. The light exits parallel with the axis. In Zone 3, the light refracts toward the focal point first and then refracts again, exiting parallel with the axis.

**Figure 9:**
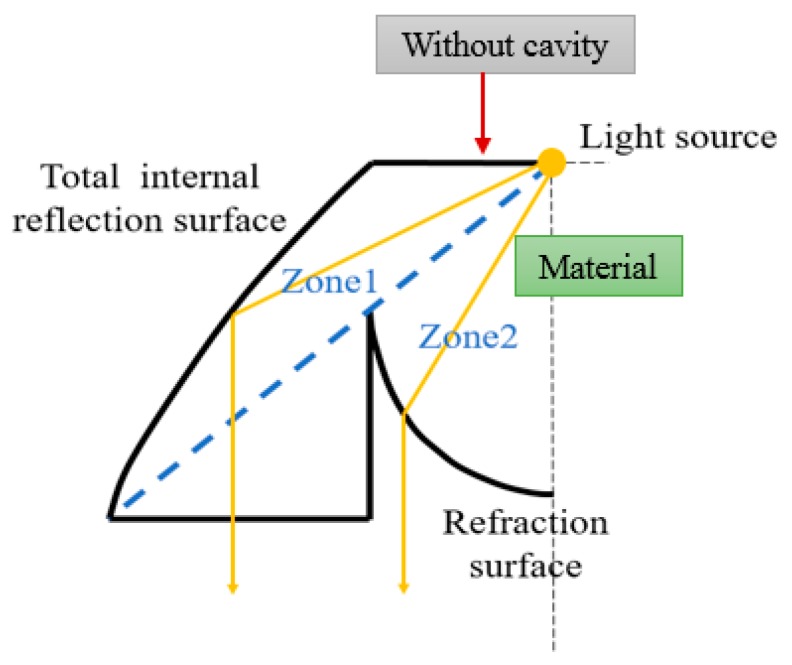
The concept of the new TIR collimator.

The third and fourth types were based on the previous collimator designs. The NACPC was placed above the collimator. If the NACPC is made from a solid structure, the light continuously goes through different mediums. This brings about unnecessary refraction and decreases the optical efficiency. Therefore, for the solid NACPC, the spherical surface was filled with the same material as the NACPC, resulting in a new TIR collimator and a new elliptical collimator. However, the concept of the new TIR collimator was the same as the original, as shown in Figure 9. The light source in Figure 7 is in the cavity of TIR, where the medium is air. When the light hits the material (entering Zones 1 and 2), it could cause slight loss through different media (from air to Zones 1 and 2). However, the light source in Figure 9 is inside the material, where the medium is the material. If the contact between the CPC and TIR collimator is perfect, there is no loss of light transmission due to the same medium.

**Figure 10:**
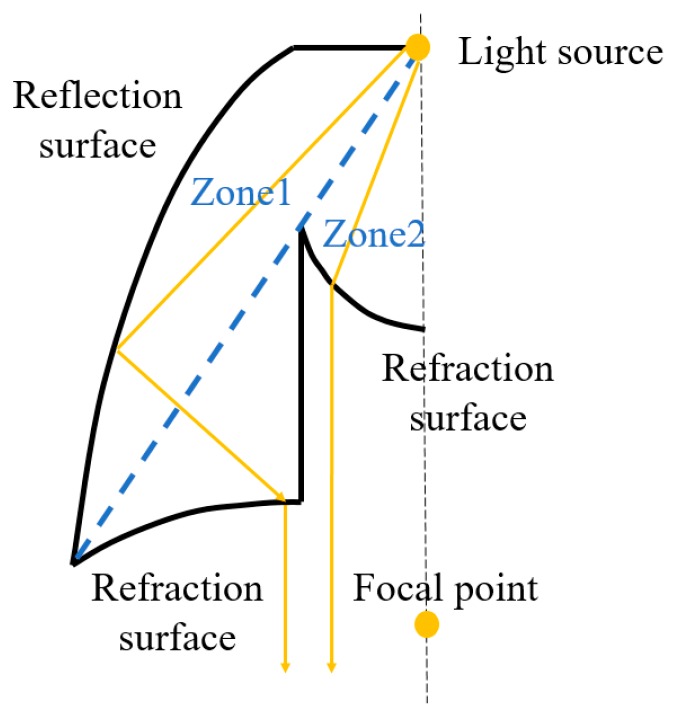
The concept of the new elliptical collimator.

The concept of the new elliptical collimator is shown in Figure 10, with two zones included in the design. In Zone 1, the light is reflected toward the focal point via the surface. Then, the light refracts and exits the new collimator in parallel with the axis. In Zone 2, the light refracts at the surface and exits parallel with the axis.

## 3. Results and Discussion

### 3.1. The Simulation Results of the NACPC

**Figure 11:**
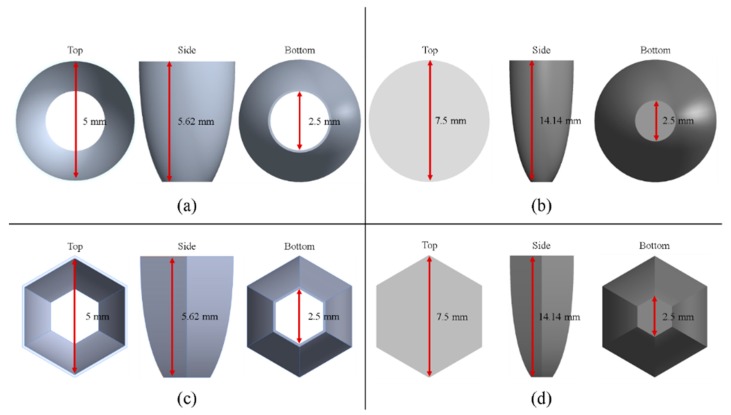
The parameters and appearances of the (**a**) mirror-CPC, (**b**) solid-CPC, (**c**) mirror-NACPC, and (**d**) solid-NACPC.

**Figure 12:**
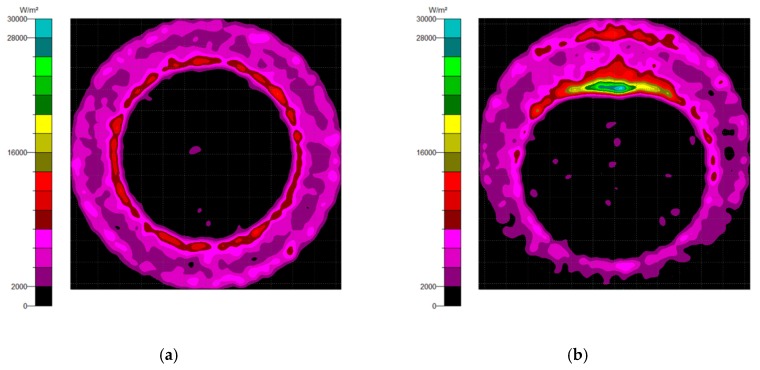

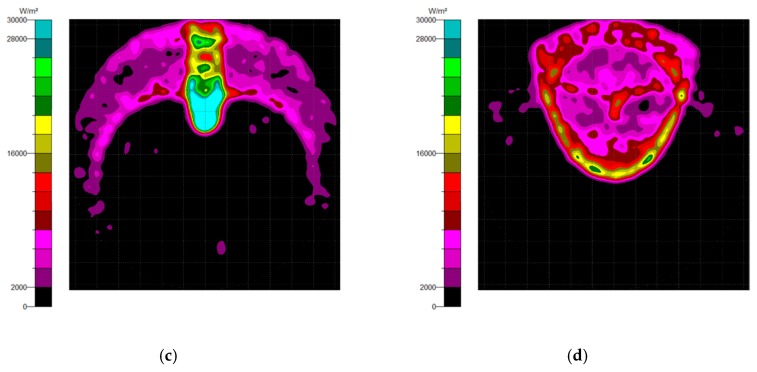
The irradiance distribution of the mirror-CPC with misalignment angles of (**a**) 0°, (**b**) 10°, (**c**) 20°, and (**d**) 30°.

**Figure 13:**
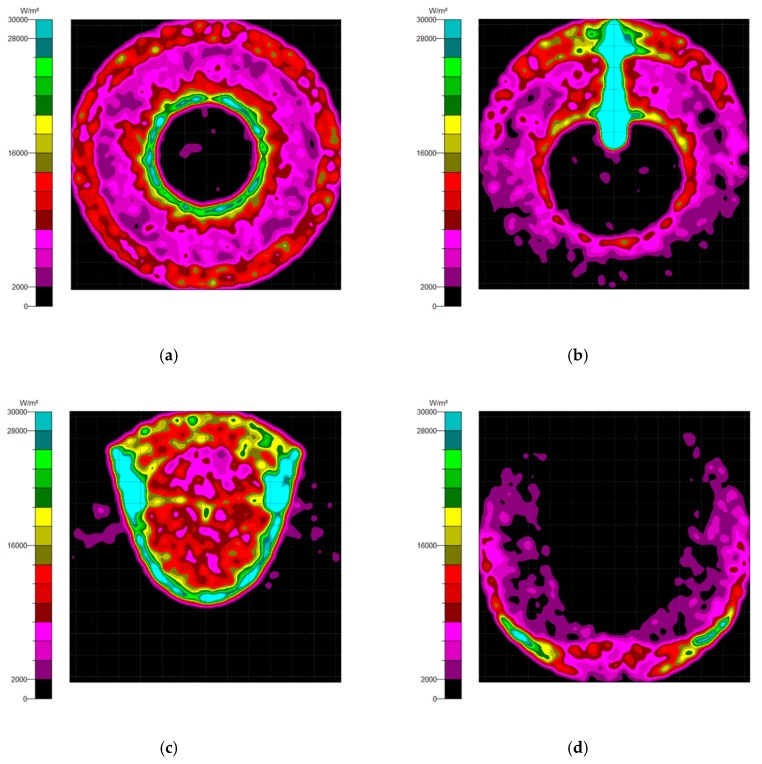
The irradiance distribution of the solid-CPC with misalignment angles of (**a**) 0°, (**b**) 10°, (**c**) 20°, and (**d**) 30°.

**Figure 14:**
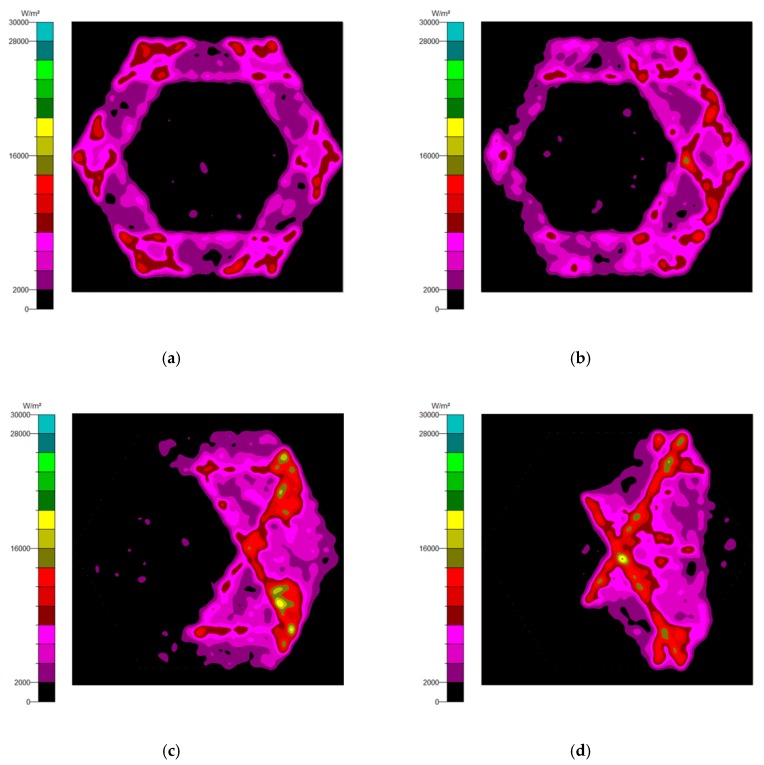
The irradiance distribution of the mirror-NACPC with misalignment angles of (**a**) 0°, (**b**) 10°, (**c**) 20°, and (**d**) 30°.

**Figure 15:**
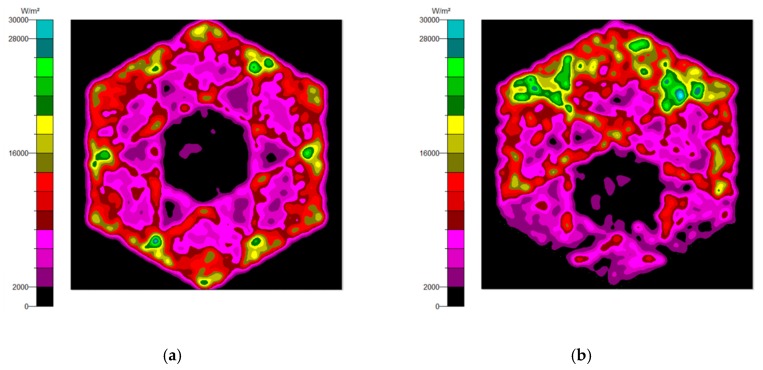

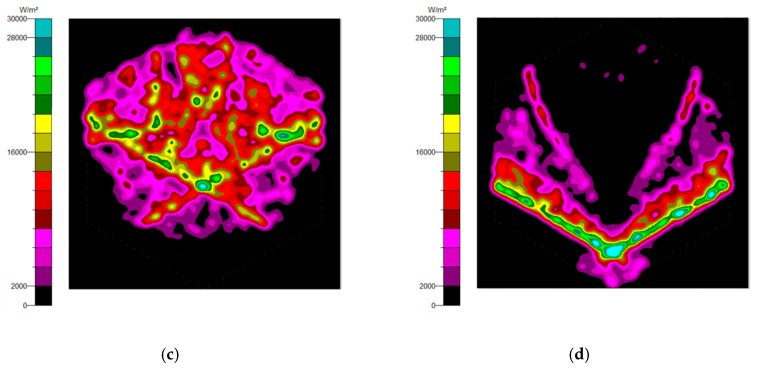
The irradiance distribution of the solid-NACPC with misalignment angles of (**a**) 0°, (**b**) 10°, (**c**) 20°, and (**d**) 30°.

Generally, there are two types of CPC: mirror and solid. The material of the solid CPC was poly (methyl methacrylate) (PMMA), with a refractive index of 1.4935. As for the shape, there are two kinds, as mentioned earlier. The CPC uses a circular aperture, while the NACPC uses a hexagonal aperture. There are four parameters used to decide the geometry of the CPC: the entrance aperture, the exit aperture, the half-acceptance angle, and the height. Two of them are decided and the other two are fixed. To explore the influence of the shape and material, the simulation was divided into four parts, mirror-CPC, solid-CPC, mirror-NACPC, and solid-NACPC. For these four groups, the fixed parameters were the exit aperture and the half-acceptance angle. The diameter of the exit aperture was 2.5 mm. The misalignment angle was fixed at 30°. In other words, the half-acceptance angle of the mirror-CPC was 30°, and the half-acceptance angle of the solid-CPC was about 19.5° due to the material. The parameters and appearances of the four groups are shown in Figure 11. To simplify the complexity of the simulation, one wavelength (546.1 nm) was applied. The simulation results of the irradiance distribution are shown in Figure 12, Figure 13, Figure 14 and Figure 15 for the mirror-CPC, solid-CPC, mirror-NACPC, and solid-NACPC at 0°, 10°, 20°, and 30°, respectively.

**Figure 16:**
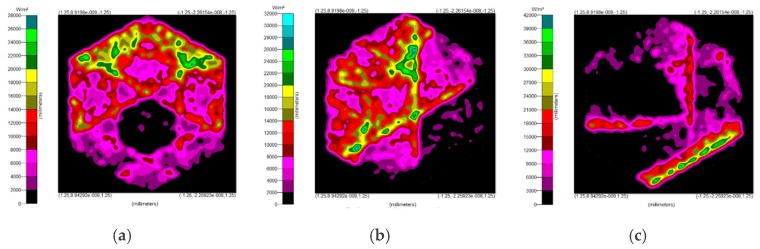
The irradiance distribution of other lateral orientations at (**a**) 0°, (**b**) 15°, and (**c**) 20°.

The results for light at 0° and 15° are shown in Figure 16, where the luminous flux was ~0.034 W and ~0.032 W, respectively. The difference between the two results at the 0° and 15° orientations was not significant. When the lateral orientation was shifted to 20° and higher, it showed a loss of about 20% or more.

**Figure 17:**
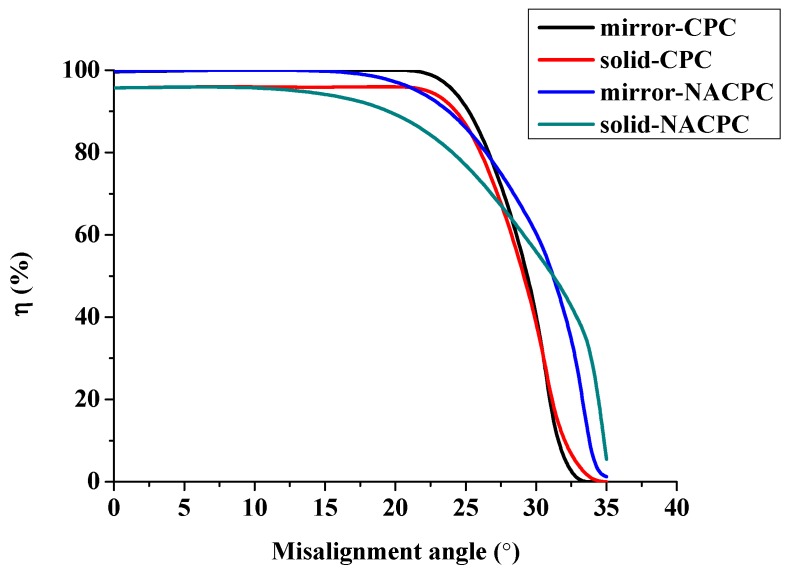
The optical efficiency of the CPC and NACPC.

The optical efficiency η is defined as the ratio of power entering and exiting the aperture. It is described by Equation (2). The optical efficiency results are shown in Figure 17. The efficiency of all the groups was maintained over 80% when the misalignment angle was smaller than 20°. The efficiency of the CPC decayed rapidly from 25° to 30°. The efficiency of the NACPC decreased earlier than that of the CPC, but the NACPC accepted a larger angle of incident light than the CPC because of its bigger aperture. The solid-NACPC was chosen for the subsequent simulations due to its machining and area utilization.

(2) $$\eta =\frac{P_{out}}{P_{in}}\times 100\%.$$

### 3.2. Simulation Results of the Freeform Surface Collimator

**Figure 18:**
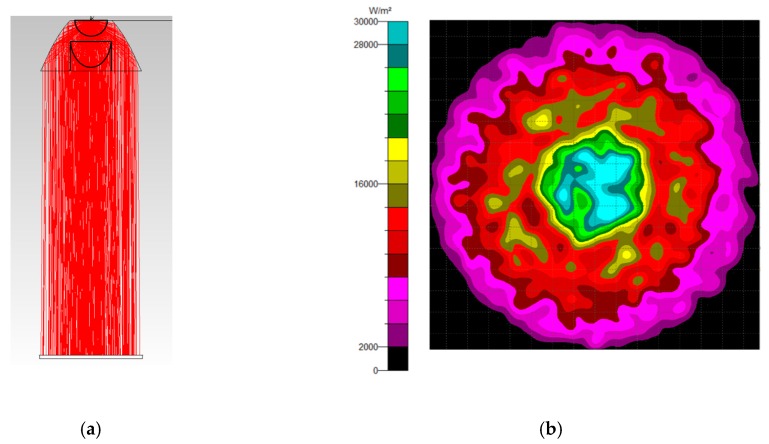

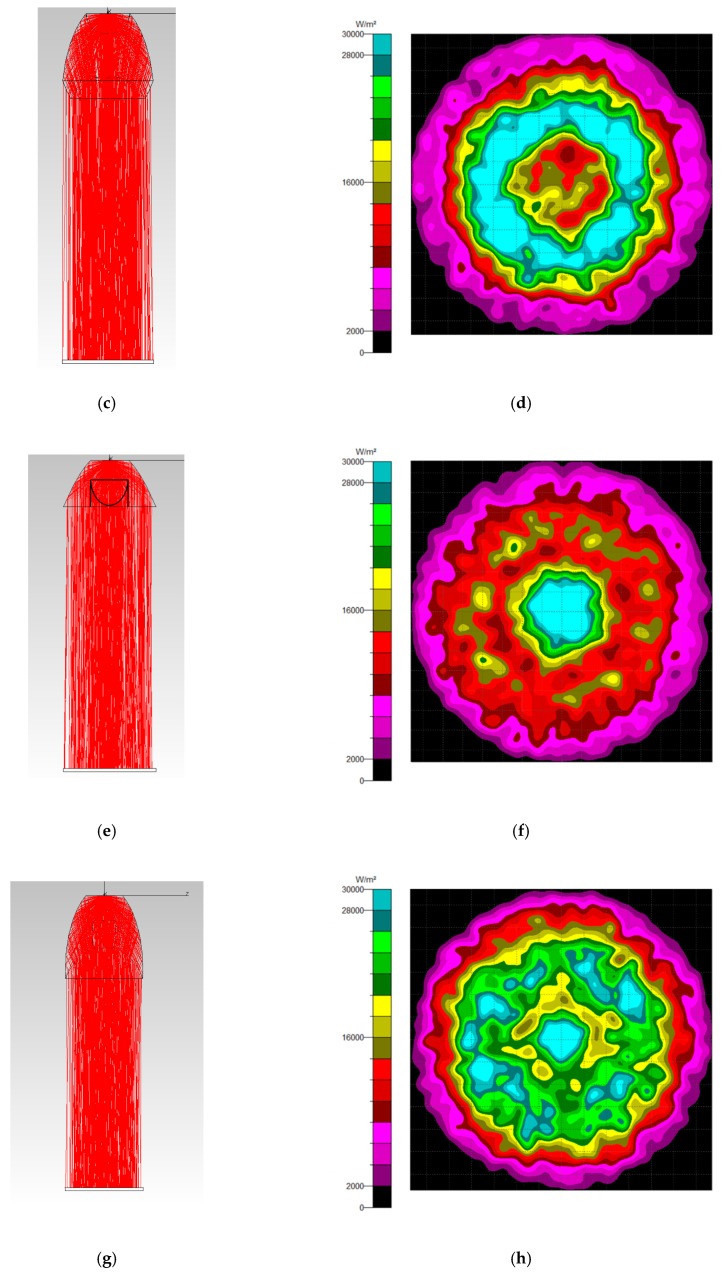
The results of the freeform surface collimators: (**a**) the ray trace and (**b**) the irradiance distribution of the TIR collimator; (**c**) the ray trace and (**d**) the irradiance distribution of the elliptical collimator; (**e**) the ray trace and (**f**) the irradiance distribution of the new TIR collimator; (**g**) the ray trace and (**h**) the irradiance distribution of the new elliptical collimator.

Using the freeform surface approach in Section 2.2, four collimators were designed. A point light source was placed at the origin point to test the performance of the four collimators. Figure 18 shows the results of the ray trace and the irradiance distribution. Each collimator aligned the light perfectly. Considering the utilization of area, the NACPC was more effective than the CPC. Therefore, the NACPC was chosen for use with the freeform surface collimator. Then, the NACPC and the freeform surface collimator were assembled to generate a misalignment light-guiding unit.

### 3.3. Analysis of the Misalignment Light-Guiding Unit

**Figure 19:**
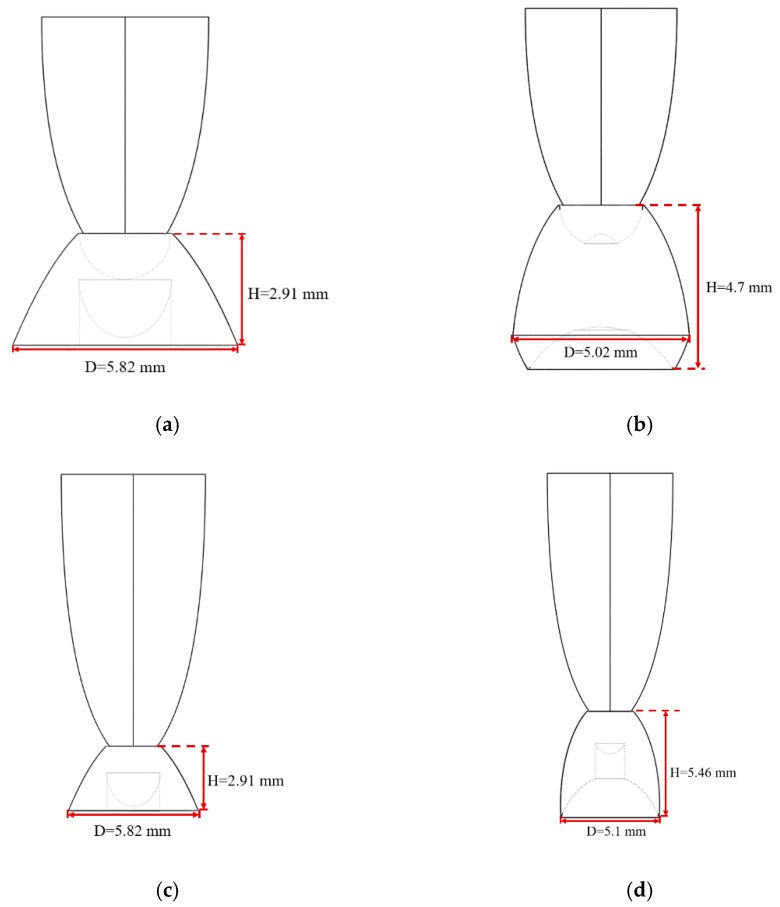
The four cases and sizes of misalignment light-guiding units: (**a**) the mirror-NACPC with the TIR collimator; (**b**) the mirror-NACPC with the elliptical collimator; (**c**) the solid-NACPC with the new TIR collimator; (**d**) the solid-NACPC with the new elliptical collimator.

**Figure 20:**
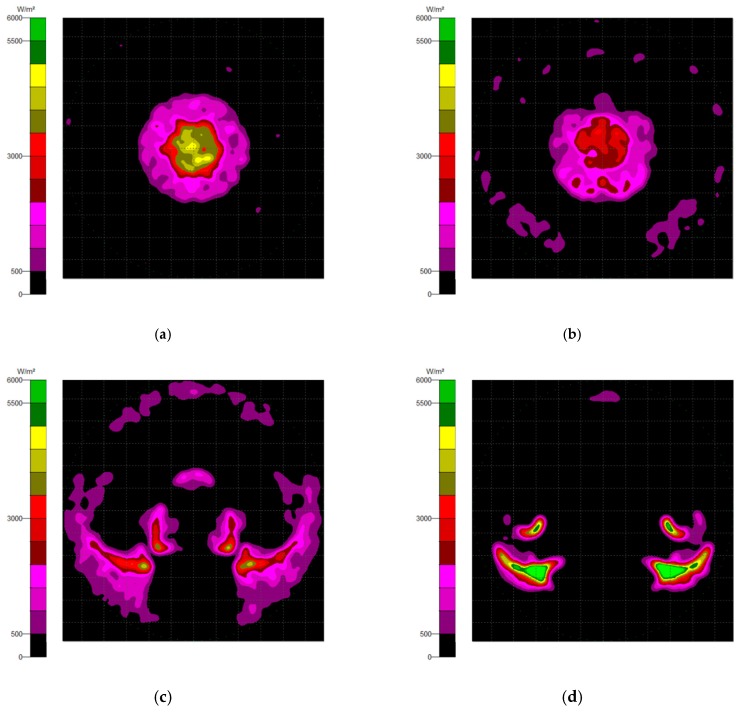
The irradiance distribution of the mirror-NACPC and the TIR collimator with misalignment angles of (**a**) 0°, (**b**) 10°, (**c**) 20°, and (**d**) 30°.

**Figure 21:**
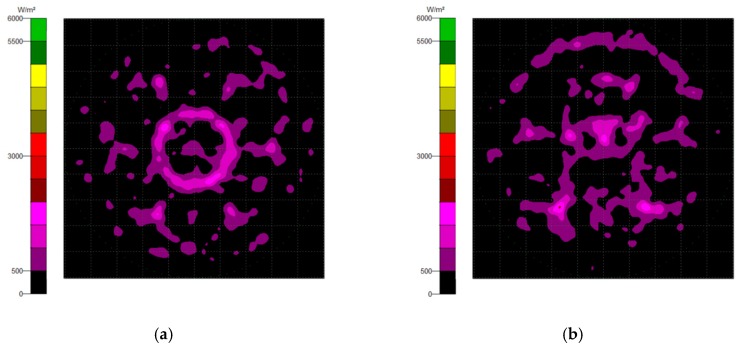

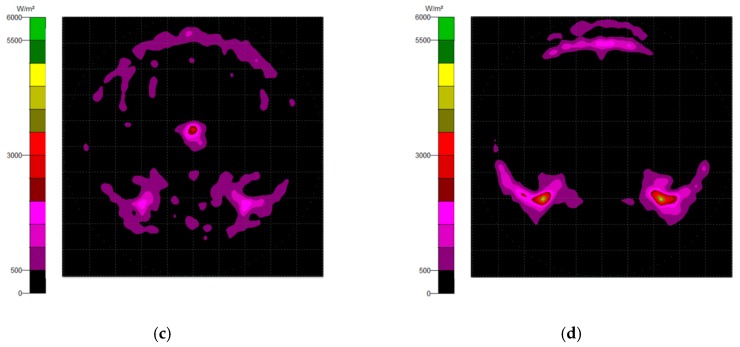
The irradiance distribution of the mirror-NACPC and elliptical collimator with misalignment angles of (**a**) 0°, (**b**) 10°, (**c**) 20°, and (**d**) 30°.

**Figure 22:**
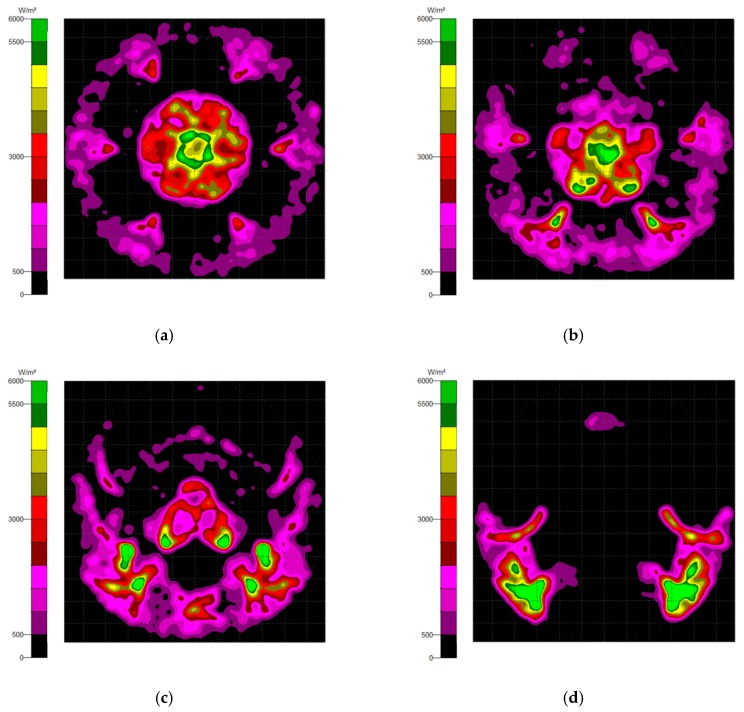
The irradiance distribution of the solid-NACPC and new TIR collimator with misalignment angles of (**a**) 0°, (**b**) 10°, (**c**) 20°, and (**d**) 30°.

**Figure 23:**
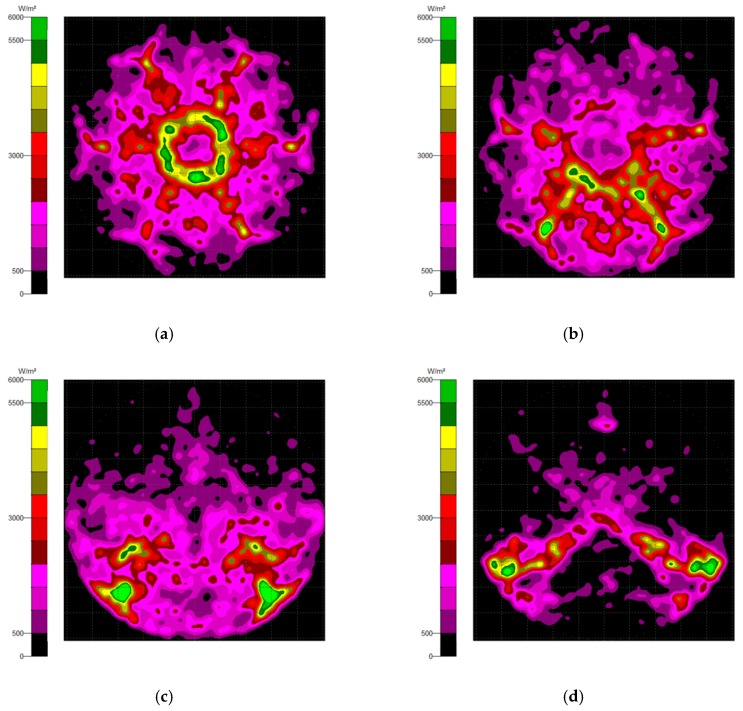
The irradiance distribution of the solid-NACPC and new elliptical collimator with misalignment angles of (**a**) 0°, (**b**) 10°, (**c**) 20°, and (**d**) 30°.

The NACPC combined with the freeform surface collimator constituted the misalignment light-guiding unit. There were four feasible freeform surface collimators, as shown in Section 3.2. Therefore, there were four cases of misalignment light-guiding unit subjected to simulation. The four cases and sizes are shown in Figure 19. These were the mirror-NACPC with the TIR collimator, the mirror-NACPC with the elliptical collimator, the solid-NACPC with the new TIR collimator, and the solid-NACPC with the new elliptical collimator. The irradiance distribution of the four cases are shown in Figure 20, Figure 21, Figure 22 and Figure 23. The misalignment light-guiding unit had better uniformity than the CPC and NACPC.

**Figure 24:**
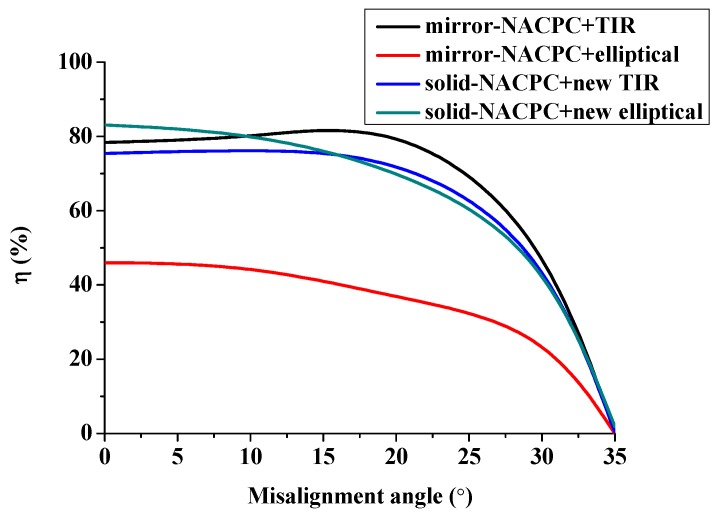
The optical efficiency of the misalignment light-guiding units.

The optical efficiency of the four cases is shown in Figure 24. The mirror-NACPC was suitable for use with the TIR collimator, and the solid-NACPC was suitable for use with the new elliptical collimator. Other than the mirror-NACPC with the elliptical collimator, the optical efficiency the misalignment light-guiding units was above 70% with a misalignment angle smaller than 20°.

The efficiency of the mirror-NACPC and solid-NACPC was good. However, taking the machining into consideration, the solid-NACPC was much more feasible than the mirror-NACPC. Therefore, the solid-NACPC was chosen for the subsequent analysis. Furthermore, the new elliptical collimator was found to have better performance than the new TIR collimator. Therefore, the solid-NACPC and new elliptical collimator were chosen for the array and subsequent experiment.

### 3.4. The Misalignment Light-Guiding Unit Array

**Figure 25:**
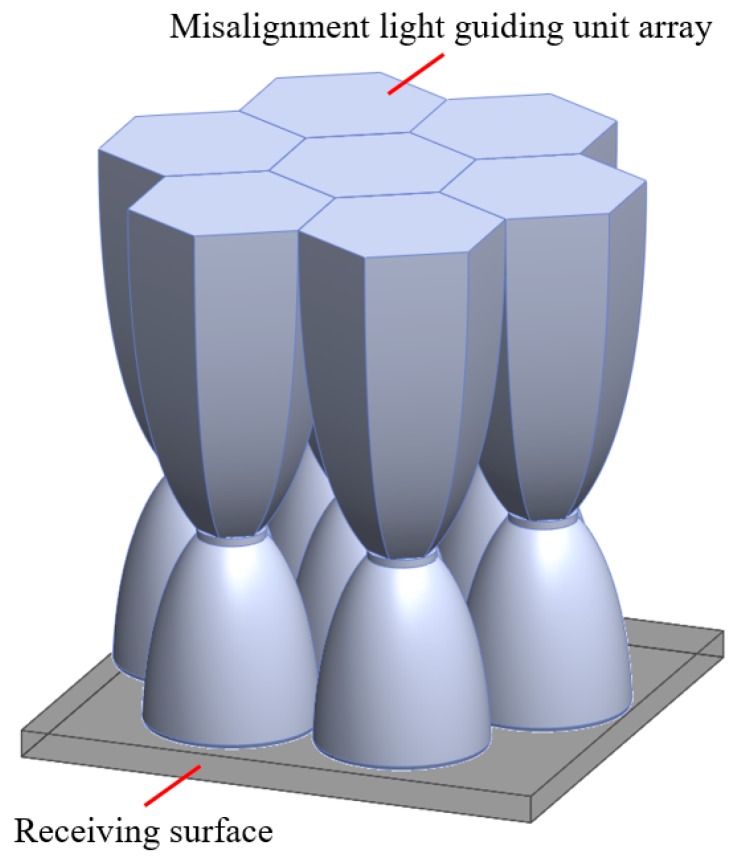
The misalignment light-guiding unit array.

**Figure 26:**
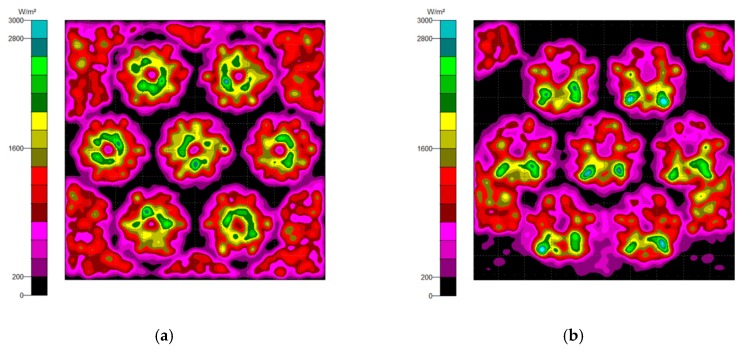

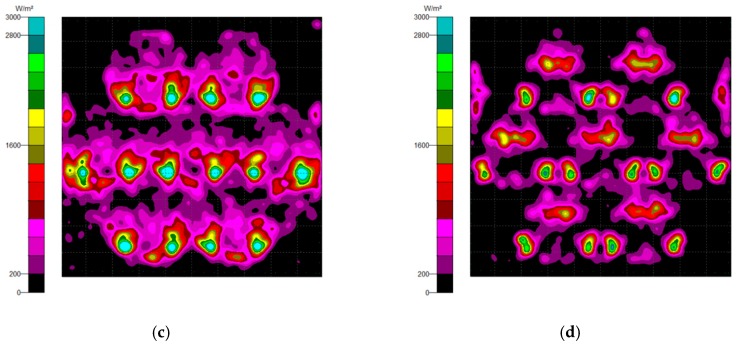
The irradiance distribution of the misalignment light-guiding unit array at misalignment angles of (**a**) 0°, (**b**) 10°, (**c**) 20°, and (**d**) 30°.

**Figure 27:**
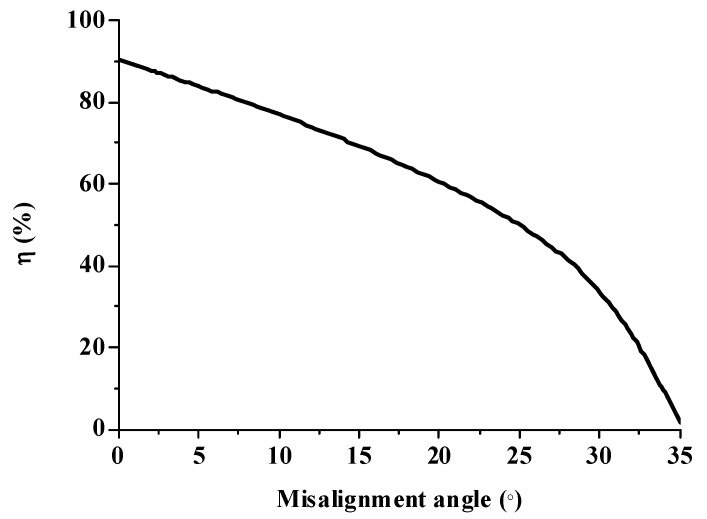
The optical efficiency of the misalignment light-guiding unit array.

The misalignment light-guiding unit was composed of the solid-NACPC and the new elliptical collimator. The unit was put in the array as shown in Figure 25. A receiving surface with an area of 15 mm × 13 mm was placed at the bottom of the array. The irradiance distribution is shown in Figure 26. The optical efficiency is shown in Figure 27. The reason for the difference in results between Figure 24 and Figure 27 is that the difference in light receiving area led to a calculation error of light collection efficiency. The array had a larger receiving square area than that of the single unit. It also contained more interspacing in the array form (i.e., a lower fill factor). In this study, the entire area of the light receiving square was chosen for the calculation of output light irradiance. The efficiency calculation was defined as the ratio of the input and output incident sun light. Therefore, a lower efficiency was achieved for the square area of the array, as it was larger than the single unit. Nerveless, the optical efficiency of the array at angles of ±30° could still reach about ~40%

### 3.5. The Misalignment Light-Guiding Unit Array

**Figure 28:**
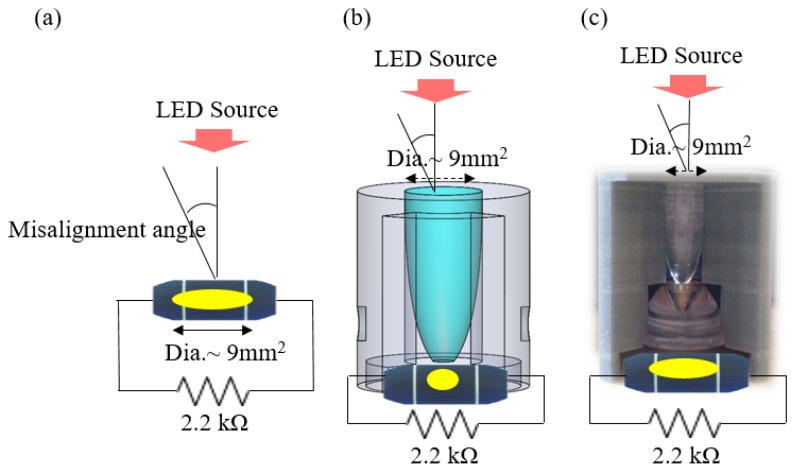
The experimental set-up: (**a**) the naked photovoltaic (PV) panel; (**b**) the CPC; (**c**) the CPC with the collimator.

**Figure 29:**
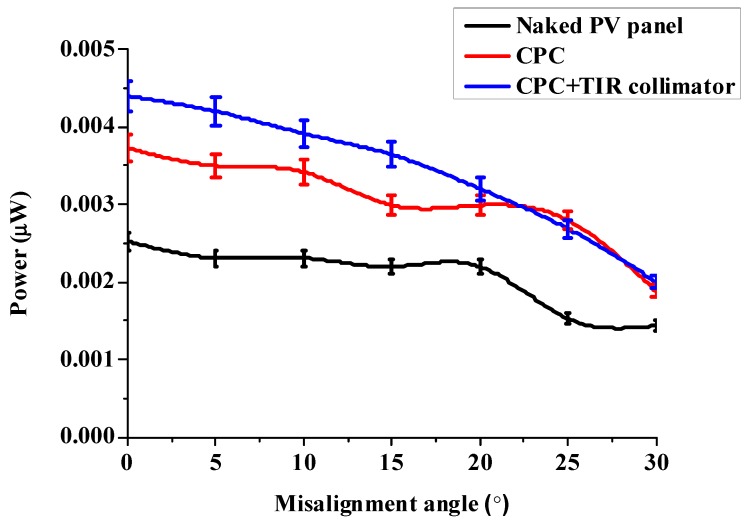
The experimental results of the CPC with the collimator.

The CPC/collimator was created and provided by the Metal Industries Research and Development Center (MIRDC, a non-profit research organization in Taiwan, Kaohsiung, Taiwan). The half-acceptance angle of the CPC was 25°. Three conditions of power generation were measured, i.e., the naked PV panel, the CPC, and the CPC combined with the TIR collimator, using the same light collection area of 9 mm^2^. All experimental set-ups had a resistance of 2.2 kΩ connected in series. The set-up is shown in Figure 28, and the experimental results are shown in Figure 29. The results showed that the CPC combined with the collimator had the largest power generation compared to the naked PV panel and the CPC. It was estimated that the CPC with the collimator had 37% higher power generation than the naked PV panel, while the CPC had 24% higher power generation.

**Figure 30:**
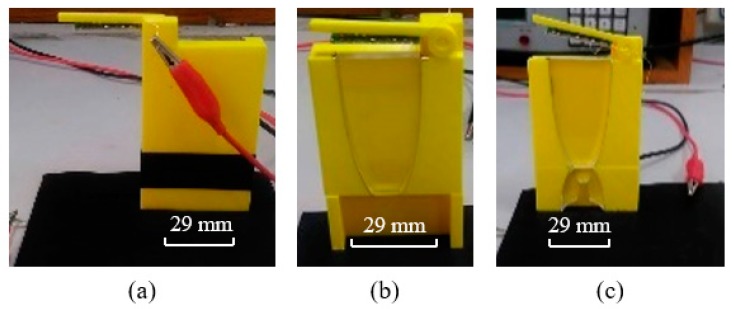
The experimental results of the two-dimensional (2D) prototype: (**a**) the naked PV panel; (**b**) the NACPC; (**c**) the NACPC with the new elliptical collimator.

**Figure 31:**
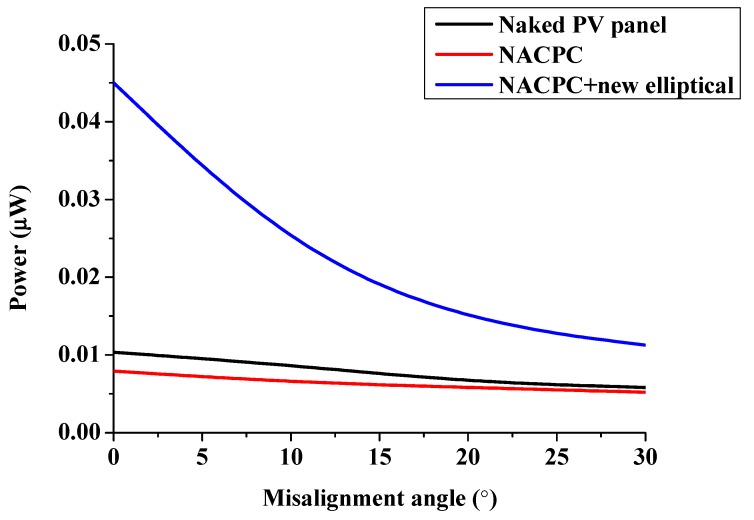
The experimental results of the 2D prototype of the NACPC with the new elliptical collimator.

After the experiment using the CPC/collimator, the two-dimensional (2D) prototype of the NACPC/new elliptical collimator was developed for the experiment via laser cutting. The material used was PMMA, and the margin had a wavy pattern after laser cutting. Therefore, the prototype was easily polished before the experiment. Three conditions of power generation were measured, i.e., the naked PV panel, the NACPC, and the NACPC with the new elliptical collimator. They all had a resistance of 2.2 kΩ connected in series, and the entrance apertures had dimensions of 29 mm × 5 mm. The yellow part shown in Figure 30 was the frame used to fix the prototype, and a rotatable light source was included above the prototype. The black paper blocked was used to block the unnecessary area. The set-up is shown in Figure 30, and the experimental results are shown in Figure 31. The experimental results showed that the power generation of the CPC was 1.8 times higher than that of the naked PV panel after calculating the area under the curve. The areas of the naked panel in Figure 28 (9 mm^2^) and Figure 29 (90 mm^2^) were different; thus, the power was affected.

## 4. Conclusions

A misalignment light-guiding module to increase the effectiveness of absorbing light was successfully investigated. The module was made up of an array of misalignment light-guiding units which consisted of an NACPC and a freeform surface collimator. The CPC can effectively collect light within the half-acceptance angle. For higher area utilization, the CPC was transformed into the NACPC. The hexagonal shape substantially increased the area utilization. The simulation results showed that both the CPC and NACPC had an optical efficiency above 80% with a misalignment angle smaller than 20°. To better align the light from the NACPC, four collimators were constructed using the freeform surface approach. Then, the NACPC was combined with the collimator to generate a misalignment light-guiding unit. The simulation results showed that the irradiance of the misalignment light-guiding unit had better uniformity than only the NACPC. The optical efficiency of the misalignment light-guiding units was above 70% with a misalignment angle smaller than 20°. The 2D experiment showed that the power generation of the misalignment light-guiding unit was 1.8 times higher than that of the naked PV panel. This module can be easily miniaturized and attached to a window or roof for micropower generation.
